# Genetic determinants of hepatitis B vaccine response

**Published:** 2011-01-10

**Authors:** Eileen Png, Anbupalam Thalamuthu, Rick TH Ong, Harm Snippe, Herawati Sudoyo, David H Muljono, Sangkot Marzuki, Greet J Boland, Mark Seielstad

**Affiliations:** 1 Genome Institute of Singapore, Singapore 138672; 2 Department of Medical Microbiology and Virology, University Medical Center Utrecht, 3584 CX, Utrecht, The Netherlands; 3 Eijkman Institute for Molecular Biology, Jakarta 10430, Indonesia; 4 Current Address: Institute for Human Genetics, University of California, San Francisco, CA 94143; and Blood Systems Research Institute, 270 Masonic Avenue, San Francisco, CA 94118, USA

## 

Hepatitis B is a major public health problem. Approximately one-third of the world’s population has serological evidence of infection with hepatitis B virus (HBV). 350 million of these are carriers who have chronic HBV infection, and about a million of these carriers die each year from chronic liver disease, including cirrhosis and liver cancer. Fortunately, HBV is a vaccine preventable disease, and the increasing adoption of this vaccine has led to dramatic reductions in the morbidity and mortality caused by this virus. However, as much as 10% of the population fails to mount a protective immune response after vaccination. Twin and other epidemiological studies have demonstrated an unusually high heritability to this trait, which suggests a high likelihood of identifying genetic variation influencing HBV vaccine response. In search of such variation, we performed a two stage Genome Wide Association Scan (GWAS) in participants of a vaccine efficacy trial from Batam, Indonesia. In Stage 1, we used the fixed content Illumina Infinium 550K SNP BeadChip to genotype 2000 vaccinees. Tests of association were performed to identify 7,000 SNPs to carry forward to a second stage of 2,300 vaccinees. Several independent regions attained genome-wide significance, including the HLA-DR and HLA-DP regions. Additional details on the study design and results will be discussed in the presentation.

